# A systematic review of cognition and functionality in delusional disorder

**DOI:** 10.1192/j.eurpsy.2021.1410

**Published:** 2021-08-13

**Authors:** H. De Diego Ruiz, M. González González, C. Martínez Díaz-Caneja

**Affiliations:** Psychiatry, Hospital General Universitario Gregorio Marañón, Madrid, Spain

**Keywords:** Delusional disorder, Systematic review, Functionality, cognition

## Abstract

**Introduction:**

Current definitions for delusional disorder (DD) state that no cognitive or functional impairment is present. However, this assumption lacks empirical validation and has been questioned by numerous authors over the years. Through systematic search we collected articles that compare patients with DD with either healthy controls or patients with schizophrenia on the basis of their cognitive symptoms and their functional outcomes.

**Objectives:**

Our aim is to draw conclusions from the available evidence on neurocognitive and functional affectation of DD.

**Methods:**

Systematic electronic search was performed using Pubmed and Embase databases. Inclusion criteria included that selected articles must be original studies, must be published in peer-reviewed journals, must contain a sample of DD patients that is compared with a sample of healthy controls and/or patients with schizophrenia and that samples must be compared on the basis of cognitive and/or functionality parameters. A qualitative synthesis was performed due to heterogeneity in data.

**Results:**

According to the information collected through our systematic review, DD patients tend to perform worse than healthy control in tests assessing cognitive functions. Results are not as conclusive regarding comparison between DD and schizophrenia, with mixed outcomes. When it comes to functionality, results are not conclusive either, with some degree of evidence pointing towards a better functioning in patients with DD in comparison to patients with schizophrenia.

**Conclusions:**

Results agree with many authors who consider both conditions as part of a psychosis spectrum. Cognitive interventions, such as cognitive remediation, must be studied for their potential role in the treatment of patients with DD.

